# Preferences on how to measure and discuss health related quality of life within integrated care for children with obesity

**DOI:** 10.1186/s41687-021-00381-3

**Published:** 2021-10-14

**Authors:** Minke M. A. Eilander, Marieke M. A. van Mil, Leandra W. Koetsier, Jacob C. Seidell, Jutka Halberstadt

**Affiliations:** 1 Department of Health Sciences, Faculty of Science, Vrije Universiteit Amsterdam, Amsterdam, The Netherlands; 2 Amsterdam Public Health Research Institute, Amsterdam, The Netherlands

**Keywords:** Children, Obesity, Quality of life, Integrated care, Implementation, Patient reported outcome measures

## Abstract

**Background:**

Childhood obesity can affect physical as well as psychosocial wellbeing. Therefore, childhood obesity care aims to improve all dimensions of health related quality of life (HRQoL). HRQoL can be measured with the generic questionnaire PedsQL4.0 and the weight-specific IWQOL-Kids. In the Netherlands, HRQoL assessment is conducted by the coordinating professional (CP). The aim of this qualitative study was to examine how and when to implement the measurement and discussion of HRQoL using the PedsQL4.0 and IWQOL-Kids within the integrated care for children with obesity in the Netherlands. Semi-structured interviews were conducted with fourteen CPs, in which the following was discussed: a) familiarity and attributions with regard to the assessment of HRQoL; b) wishes and needs with regard to the usage of the questionnaires; c) its practical incorporation.

**Results:**

Interviews revealed that most CPs gained insight into the HRQoL by talking with families. One CP used the PedsQL4.0, the remaining CPs were unfamiliar with the two questionnaires. Even though some barriers, for instance a lack of time, might hinder the implementation of the PedsQL4.0 and IWQOL-Kids, all participants think the usage of either one or both questionnaires would have additional value to the support and care for children with obesity. There was no consensus about the questionnaire of preference.

**Conclusions:**

When the right preconditions are met, HRQoL questionnaires have the potential to support CPs in improving the care for children with obesity, tailored to each individual child.

## Introduction

Over the past decades, the prevalence of obesity among children has increased globally [1]. Currently in the Netherlands, about 2–3% of the children 2–18 years old are estimated to have obesity [2], which challenges their physical and mental health and their physical and psychosocial wellbeing [3, 4]. Since obesity is primarily caused and sustained by behavior that is driven by an interaction between biological, psychological and environmental factors, these factors should be assessed in children with obesity seeking support and care [5]. In the Netherlands, the importance of support and care for these children is well understood and preconditions are made to facilitate this, which is quite uncommon in other countries [6].

Currently in several municipalities in the Netherlands the national model Integrated Care for Childhood Overweight and obesity (ICCO) (0–19 years) is going through an implementation process. This model provides a basis for the local integrated support and care for these children. The model includes a six-step trajectory in order to provide support and care [7]: (1) ‘diagnose obesity’, (2) ‘conduct a broad assessment’, (3) ‘discuss the interrelatedness of factors and determine which approach to take’, (4) ‘design a care plan that includes agreements with the child, the parents and other professionals’, (5) ‘begin to execute the care plan’, and (6) ‘make sure the resulting changes are sustained’. An important role within this model is the coordinating professional (CP), which is often fulfilled by a youth health care (YHC) nurse who received additional training in order to work as a CP. He or she coordinates the support and care provided by various professionals, stimulates and encourages the child and its primary care givers (in most cases parents), monitors the progress, and initiates any follow-up steps that may prove necessary.

To understand the situation of each individual child and parent, step 2 (‘conduct a broad assessment’) is key in the model, and consists of a physical examination (including the determination of obesity and the weight-related health risks) and the assessment of psychosocial wellbeing, family’s circumstances and their lifestyle behavior. This second part is often conducted by the CPs themselves. The information gained by the broad assessment serves as starting point for the treatment plan and suitable intervention(s). The ultimate goal of the support and care for these children is to improve their health, societal participation and health related quality of life (HRQoL) in the short and long run by adopting a healthier lifestyle and to help them sustain the behavior change.

Since the national model ICCO has a person centered approach, the usage of Patient Reported Outcome Measures (PROMs) is key, in which physical, emotional, and social domains of life are addressed [8, 9]. HRQoL questionnaires are examples of PROMs. HRQoL is defined as ‘the way health affects quality of life’ [10]. The latter is defined by subjective indicators collectively reflecting a broad range of life domains [11]. The HRQoL of children with obesity can be affected on several domains [4, 12–16], such as physical functioning, social life, emotional wellbeing, body esteem and school functioning [13, 17]. When Body Mass Index-Z scores increase, average HRQoL decreases [14, 18]. Social support, high self-esteem and good emotional wellbeing are found to be positive predictors of better HRQoL in children with obesity [19] and therefore seem worthy to strengthen within an intervention. The HRQoL of these children can be improved by a combined lifestyle intervention [20], even if the weight loss is partially regained [21].

The assessment of HRQoL can serve three purposes: firstly, as a diagnostic assessment; secondly, to guide a tailored and targeted treatment; thirdly, to evaluate the outcome of the treatment. Therefore, the assessment of HRQoL of children with obesity is recommended in (inter)national guidelines [22]. In order to assess HRQoL, previous research [23, 24] concluded that the general HRQoL questionnaire ‘Pediatric Quality of Life Inventory 4.0’ (PedsQL4.0) [25] and the weight-specific HRQoL questionnaire ‘The Impact of Weight on Quality of Life for Kids’ (IWQOL-Kids) [26–28] seem suitable options for this specific population in the Netherlands. Both questionnaires have a Dutch child and parent proxy version and are digitally available for CPs within the context of the national model ICCO [29]. By visiting an online webtool [30] children or their parents complete the PedsQL4.0, the IWQOL-Kids or both online and the outcomes are send to the professional. The outcomes are discussed with the child and/or parents(s).

Although the importance is well understood, research shows that practical barriers (such as professionals’ lack of time to organize facilities required for assessment, to actually assess HRQoL and to discuss the outcomes with the child or family) hinder the application of HRQoL assessments [31]. Hence, recommendations on how to practically incorporate the measurement and discussion of HRQoL within the national model ICCO might stimulate the assessment. Therefore, the current study aims to answer the question: how and when can the measurement and discussion of HRQoL with the usage of the PedsQL4.0 and IWQOL-Kids be practically incorporated within the national model ICCO?

## Methods

### Procedure and participants

This study was approved by the medical ethical committee of VU University Medical Center (METC number 2019.511) and COREQ criteria were taken into consideration [32]. Semi-structured, open-ended, face-to-face interviews with CPs were conducted. Interviewers (M.v.M. & L.K.) were trained in qualitative research methods. The interview topic list was pilot tested prior to the interviews (M.E.). Two project leaders from two municipalities and 25 CPs from nine different municipalities were approached face-to-face, by telephone or by email to recruit participants. The interviews were audio recorded.

### Measures

Prior to the interviews, participants were asked to visit the online webtool to gain some insight into the webtool, the PedsQL4.0 and the IWQOL-Kids. The PedsQL4.0 consist of 23 items scored on a 5 point Likert scale reflecting upon the child’s physical, emotional, social and school functioning (e.g. “It’s hard for me to walk more than one block”, “Others tease me”, “I miss work or school to go to the doctor or hospital”) [25]. The IWQOL-Kids consist of 27 items scored on a 5 point Likert scale reflecting upon the child’s physical comfort, body esteem, social life and family relations (e.g. “Due to my weight, I’m ashamed of my body” “Due to my weight, people talk behind my back”, Due to my weight, it’s hard to move around”) [26].

The following topics were addressed in the interviews with the CPs: a) familiarity and attributions regarding the measurement and discussion of HRQoL by the usage of the PedsQL4.0 and IWQOL-Kids (with or without webtool) within the setting of the national model ICCO; b) wishes and needs with regard to the usage of the PedsQL4.0 and IWQOL-Kids in order to meet the three purposes of assessing HRQoL; c) wishes and needs with regard to the practical incorporation of the measurement and discussion of HRQoL within (the six steps of) the national model ICCO. After the interview, each participant received a summary of the interview to make comments and corrections if needed for the purposes of member checking.

### Analysis

Using MAXQDA version 2020, template analysis was used to analyze the interviews. Based on the interview topics a list of codes (template) was conducted representing themes identified in the textual data. While reading and analyzing the data, the template was complemented with open coding, was modified and refined when applied to further data. [33]. Interviews were transcribed verbatim. Key words and codes to extract content from the text were assigned by two researchers (M.v.M. & M.E.). Discrepancies were discussed within the research team until consensus was reached.

## Results

### Participant characteristics

Fourteen CPs from nine different municipalities were interviewed, their characteristics are presented in Table 1*.* A lack of time, personal circumstances and the participation of a colleague were reasons for CPs to decline participation in this study. Sixteen interviews were planned, however, after thirteen interviews no new information was retrieved, which was confirmed with the 14th interview. Therefore, data saturation was achieved. The majority of participants fulfilled the role of CP as YHC nurse and involvement with ICCO within this role ranged from few weeks to eight years, on average 2.2 years. The results are summarized in Table 2.

**Table 1:** Participant characteristics

	N	x̄, Sd (range)
Female / male	13/1	
Age		48.4, 11.3 (26-63)
Years of experience in current function		16.1, 10.4 (0.5-33)
Years of OCCI^a^ within own municipality		3.6, 2.5 (0.25-10)
Months of involvement in ICCO with role as CP^b^		26.8, 27.2 (1-96)
Discipline		
YHC nurse^c^ 0-12 years	2 (14%)	
YHC nurse 0-19 years	7 (50%)	
YHC nurse 12 year and older	1 (7%)	
YHC nurse 4-12 years	1 (7%)	
YHC nurse 4-19 years	2 (14%)	
Lifestyle coach	1 (7%)	

^a^Integrated Care for Childhood Overweight and obesity

^b^Coordinating professional

^c^Youth health care

**Table 2:** Summary of results

Interview topics	Main findings
(a) Familiarity and attributions regarding the measurement and discussion of HRQoL by the usage of the PedsQL4.0 and IWQOL- Kids (with or without webtool) within the national model ICCO^a^	*Current HRQoL assessment and familiarity:*
	•HRQoL was considered important
	•CPs gained insight into children’s HRQoL by talking with families
	*Attributions:*
	•CPs were unfamiliar with the questionnaires^b^
	•The questionnaires could concretize, examine and track children’s HRQoL
	•CPs appreciated the layout of the webtool
	•The questionnaires are difficult for some children
	•The questionnaires focus on limitations instead of strengths, the latter is more in line with CPs’ positive approach
(b) Wishes and needs with regard to the usage of the PedsQL4.0 and IWQOL-Kids in order to meet the three purposes of assessing HRQoL	•CPs were willing to test the webtool
	*Aims of the questionnaires according to CPs:*
	•To diagnose
	•To target treatment
	•To evaluate treatment
	•To motivate families
	•To motivate CPs to discuss HRQoL
	•The questionnaires capture children’s own answers
	•Children’s answers could help to discuss HRQoL in detail
	*Barriers for assessment:*
	•Content of the questionnaires (both: difficult and negative tone, IWQOL-Kids: confronting)
	•Lack of time
	•Additional administrative workload
	*Preconditions of assessment:*
	•Beneficial for families
	•Contribution to CPs tasks and work satisfaction
	•Enough available time
	•Additional information about the questionnaires and webtool
	•Facilitated by own organization
(c) Wishes and needs with regard to the practical implementation of the measurement and discussion of HRQoL within (the six steps of) the national model ICCO	•First assessment in step 2 (‘conduct a broad assessment’) within the national model ICCO
	•Follow up assessment between step 5 (‘start with the execution of the care plan’) and step 6 (‘make sure the achieved changes are sustained’)
	•Exact timing and place of assessments tailored to individual cases
	•Important to discuss outcomes

^a^ PedsQL4.0 and IWQOL-Kids

^b^ Integrated Care for Childhood Overweight and obesity

#### (a) Familiarity and attributions regarding the measurement and discussion of HRQoL by the usage of the PedsQL4.0 and IWQOL-Kids (with or without webtool) within the national model ICCO

All interviewed CPs mentioned that children’s HRQoL is one of the cornerstones in their work and according to five CPs, HRQoL has greater value than weight status. CPs gave a broad range of HRQoL definitions varying from children’s emotional state of mind, having friends, the ability to exercise, the atmosphere at home and in the classroom, and to some degree the financial situation. Two CPs explicitly mentioned the physical health status of the child. The majority of CPs emphasized the subjectivity of HRQoL.

This is actually about feeling well in all areas, physical, psychological and social.—Participant #12

Prior to the interviews, almost all participants were unfamiliar with the PedsQL4.0 and/or IWQOL-Kids and all but one did not measure HRQoL. This one participant used the PedsQL4.0 to evaluate the treatment. The other thirteen CPs mentioned to gain insight into the children’s HRQoL by talking with them about their lives and sometimes by observing their behavior during consultation. The use of other questionnaires (e.g. the Strengths and Difficulties Questionnaire) that tap into the social or emotional wellbeing (as part of a general youth assessment) gave additional understanding of the HRQoL, according to eleven CPs. Six CPs did not prefer to use questionnaires in general; they felt that routine conversations gave them a fair indication of the child and family situation on its own.

No, I do not do it by the means of a questionnaire. Just by telling, asking and naturally you will come across all kinds of topics – Participant #2

However, most participants explicitly mentioned that HRQoL questionnaires could serve as tools to concretize, examine and track the HRQoL. According to seven CP’s it would have an additional value to their current assessment.

Well, I think that eventually it will provide more information and maybe you will be able to better connect with what they [the children] need. That, I believe- Participant #9

The webtool was visited by all CPs prior to the interviews, by some more exhaustively than others. In general, their impressions were positive. They mentioned the accessible and understandable layout of the questionnaires, and the insightful representation of HRQoL outcomes they received after completion. Two CPs mentioned that the questionnaires might be too difficult for children in general, half of the CPs mentioned that the questionnaires are not or less suitable for families whose Dutch is not their primary language or whose literacy is less developed, therefore it was suggested that the addition of smileys or pictograms to the questions would increase the understandability. According to some CPs the tone of the questionnaires was negative due to the focus on potential limitations, which was not in line with CPs positive health approach; they preferred to focus on the abilities of the child and parents instead of the limitations and problems. With respect to the IWQOL-Kids because of its focus on weight-related limitations and problems, for children these topics might be confronting (N = 7) or unpleasant (N = 4).

Obviously in our line of work we pay attention to what you [the child] can do. (…) Our approach is very positive. And this line of questioning is.. well ‘this I can’t do and that I can’t do, and I am in bad shape’, so I can imagine that during completion this does not motivate. Or when you do not feel good about yourself, answering does not have a positive effect.- Participant #7

#### (b) Wishes and needs with regard to the usage of the PedsQL4.0 and IWQOL-Kids in order to meet the three purposes of assessing HRQoL

All interviewed CPs were willing to test whether they could incorporate the questionnaires in their routine care in the nearby future (with the usage of the webtool) for diagnostic purposes (N = 6); to target treatment (N = 12); to evaluate treatment (N = 13); to motivate children and parents into a healthier lifestyle (N = 4); and to motivate themselves to discuss HRQoL topics (N = 2). With regard to the content of the questionnaires, some CPs mentioned that the questions could serve as an opening to discuss HRQoL related topics in more detail. Furthermore, the questionnaires would capture answers given by the children themselves, instead of their parents’ or CPs’ own interpretation, and the answers would give children and parents additional insight into their own lives and wellbeing (N = 3).

CPs mentioned several possible barriers for inclusion of the questionnaires in their routine care: obstacles regarding the content of the questionnaires; a lack of time to prepare, explain, measure and discuss (the outcomes of) the questionnaires (time they rather spent interacting with the families); and the additional administrative workload of using the questionnaires. Consequently, a number of preconditions to applicate the questionnaires were mentioned: the assessment of HRQoL should be an addition for children and parents too (N = 8); it has to contribute to the CPs’ tasks and job satisfaction (N = 7); there should be enough available time per family (N = 6); there should be additional information about the purpose, the how and when, and suggestions on how to discuss the outcomes should be made available (N = 5); and it should be facilitated by the CPs organization (N = 2).

It must not increase our work load.. You must not tell us ‘Can you keep track on this and that’ – Participant #3

#### (c) Wishes and needs with regard to the practical implementation of the measurement and discussion of HRQoL within (the six steps of) the national model ICCO

There was no agreement on the preferred questionnaire: three CPs preferred to use the IWQOL-Kids as the questions are weight-specific and address topics they did not yet discuss in their conversations (e.g. the avoidance of activities in which shorts or swimsuits are required), five CPs preferred the PedsQL4.0 as they thought the tone of the questions was more appropriate, and three preferred to use both. The majority of CPs wanted to use the questionnaires in step 2 (‘conduct a broad assessment’) within the national model ICCO. The follow up measurement in order to evaluate the treatment and/or progress, should take place between step 5 (‘start with the execution of the care plan’) and step 6 (‘make sure the achieved changes are sustained’). However, the majority of CPs added that the exact timing and place of the HRQoL assessment needs to be tailored to each individual case (N = 8). CPs suggested that children or parents could complete the questionnaire at home or during the consultation with the CP, either alone or within CP’s company (when for example assistance is required). Five CPs suggested that children or parents complete the questionnaire during the consult with the CP (e.g. when the CP talks with the parents, the child can fill out the questionnaire or vice versa). The majority of CPs preferred the webtool over pen and paper.

So, a child completes it [the questionnaire] (…) and then you can have a look ‘hey are there topics that stand out’ and you can focus on those during the conversation. – Participant #5

The importance to discuss the outcomes of the questionnaires with the children and/or parents was mentioned by almost all participants. The outcomes could serve as a conversation tool to talk about HRQoL in more detail. None of the interviewed CPs felt uncomfortable to discuss the outcomes. However, two CPs felt less certain when results indicate a poor HRQoL. The worry to demotivate the child in such cases was expressed by one CP. When asked, most participants thought their communication skills and those of their colleagues to discuss outcomes were sufficient, a few mentioned that colleagues might find this difficult.

Look, if you use it [the questionnaire], you have to assume that you discuss it too. Because otherwise you don’t need to administer the questionnaire. It’s a must. That is my job. Because I believed that the questionnaire was necessary at that time.- Participant #12

## Discussion

The aim of this study was to gain insight into the how and when of the measurement and discussion of HRQoL with the usage of the PedsQL4.0 and IWQOL-Kids within the national model ICCO, according to CPs. This to provide recommendations that might stimulate the assessment and discussion of HRQoL of children with obesity.

In the interviews the CPs disclosed that HRQoL is one of the cornerstones in their work, with families although almost none of the interviewed CPs used questionnaires to assess the HRQoL of children with obesity. CPs mentioned that HRQoL questionnaires could serve as objective tools to examine and track HRQoL, they appreciated the accessible and understandable layout of the webtool. However, CPs mentioned a lack of time to prepare, examine and discuss the questionnaires. Also, the questionnaires might be too difficult for children and parents whose Dutch is not their primary language or whose literacy is less developed, and the negative phrasing or content, especially in the IWQOL-Kids, is not in line with CPs more positive approach. To our knowledge, whether the type of phrasing and content of the IWQOL-Kids affects children, has not yet been documented. In the development of a HRQoL measure it is recommended to include qualitative input from the target population, which has not been done while developing the IWQOL-Kids according to a comparison study [9]. Within that study on PROMs both the PedsQL4.0 and IWQOL-Kids scored high on negative content and phrasing, making a proper introduction and a discussion after completion necessary [9]. With respect to the usage of the questionnaires within the national model ICCO, it is recommended that this introduction should state why the questionnaire is relevant for the child and/or parent given their own personal situation [34].

CPs mentioned that the outcomes should be discussed with children and/or parents. The benefits of talking about the answers are well understood in scientific literature: firstly, it confirms or refutes the answers; secondly, it shifts the primary focus from body weight to its impact on the daily lives of the child and therefore potentially provides information for subsequent treatment options [35]; thirdly, it facilitates an autonomy-supportive and person-centered conversation and strengthens the relationship between the professional and the child and its parents [8]; fourthly, it can have a positive effect on the child’s emotional, psychosocial and physical wellbeing [36, 37]. Furthermore, the discussion of results provides an opening to intervene on potential feelings of sadness after completion. Within this conversation, CPs have the opportunity to focus on the child strengths in addition to problems and limitations, in line with the CPs’ desire for a positive approach. During this conversation a sensitive attitude is highly recommended, especially while addressing the child’s weight [38] since weight-related stigmatization also occurs in health care professionals [39]. Previous research showed that professionals’ stigma and negative attitudes with respect to obesity result in fewer assessments of HRQoL [17].

During completion, the availability of assistance might help children or parents when they have trouble understanding the questionnaires, especially when Dutch is not their primary language or when their literacy is less advanced[40]. Previous research showed that the extent to which clinicians believe that language barriers or cultural sensitivity issues hinder families to understand the content of questionnaires, results in less frequent assessment of PROMs among these families [41, 42]. Therefore, not only available assistance, but also insight into CPs’ own believes, attitudes and potential weight-stigma might contribute to more frequent data collection.

All interviewed CPs were willing to try to include the questionnaires in their routine care if they would have enough time and if it was facilitated by their organization. This willingness is the first step of a successful implementation process [43]. However, it has been shown that the embedding of HRQoL questionnaires in clinical practice is difficult, even though intentions are positive [31, 43]. CPs’ whish for available time and a stimulating work environment does not come as a surprise; concerns that the process will be time-consuming, that it interferes with the usual work flow and that questionnaires are too long for patients to complete are found in other studies too [31, 44]. Hence, the availability of a driving force (e.g. a colleague, organization, program or intervention that prioritizes HRQoL) who takes lead and responsibility of the facilitation and organization of such an environment is suggested [31, 34]. Although in some cases the discussion of HRQoL questionnaires does not lengthen the consultation duration [45], often the proceedings to organize the assessment are considered time consuming [31, 43]. Therefore, additional time facilitated by the organization can contribute to the usage of HRQoL questionnaires. From a global perspective, the Dutch situation in which the support and care for children with obesity has an integrated approach, is quite exceptional and supports the embedding of HRQoL questionnaires. In many countries treatment for children with obesity is not yet sufficient, which makes the measurement and discussion of HRQoL even more challenging [6]. In the Netherlands, an increasing amount of municipalities work according to the national model ICCO. This provides an opportunity to stimulate the usage of HRQoL measures and to integrate the recommendations mentioned. However, almost all interviewed CPs were unfamiliar with the PedsQL4.0 and IWQOL-Kids prior to the interviews. Future research is needed to evaluate if the measurement and discussion of HRQoL is indeed integrated in the routine care of CPs. For future usage of the webtool, the addition of the PedsQL4.0 and the IWQOL-Kids in different languages and questionnaires focusing on children’s strengths is recommended.CPs thought that the usage of the questionnaires could serve as additional diagnostic instruments in step 2 of the national model ICCO, to target treatment and to evaluate treatment between step 5 and step 6. It can be argued that frequent assessments monitor HRQoL more efficiently, however, additional assessments can be burdensome for children and CPs. Therefore, it may be appropriate to examine HRQoL less frequently in generally healthy and happy children and to increase the frequency of assessment in more challenging cases, including cases where the child has an increased weight-related health risk [46].

Both generic (such as the PedsQL4.0) or disease specific questionnaires (such as the IWQOL-Kids) have pros and cons: the PedsQL4.0 allows comparisons to normative populations, but may not be sensitive to changes over time, and the IWQOL-Kids is more sensitive to specific symptoms, but may miss domains that affect the child but are unrelated to obesity [46]. The various ideas on the questionnaire of preference and the preferred time and place of completion highlight the importance of a tailored approach for each individual child and/or parent.

### Limitations

Although this study provides useful information on how to implement HRQoL within the national model ICCO, some limitations deserve to be mentioned. Given the qualitative nature, the transferability of the results can be questioned. We did, however, interview CPs from different municipalities, representing both urban and rural areas. It is possible that the fourteen participants were more involved in the support and care for children with obesity and more motivated to fulfill their role as CP, than CPs not participating in the study, therefore there might be a selection bias. Prior to the interviews, some CPs completed both the IWQOL-Kids and the PedsQL4.0 on the webtool, others just visited the webtool. Consequently, certain CPs were able to give detailed insights into their wishes and needs with regard to the questionnaires, the webtool and its embedding in the national model ICCO, while others provided more general information. Some CPs were involved in ICCO for a few years (max 8 years), while others just started (a few weeks). These differences provided a great diversity of information, which is a strength. However, to establish more knowledge about the embedding of HRQoL within the national model ICCO, this study would have benefited from interviews with a larger number of seasoned CPs. The main aim of assessing HRQoL by using the PedsQL4.0 and IWQOL-Kids with the usage of the webtool is to improve the support and care for children and their parents. The degree to which children and parents believe it is beneficial for their care is not studied within this research. Future research is needed to investigate how children and parents perceive the measurement and discussion of the HRQoL questionnaires within the context of the national model ICCO.

## Conclusion

When the right preconditions are met, the HRQoL questionnaires (by the usage of the webtool) have the potential to support CPs in improving the care for children with obesity.

## Data Availability

All data generated or analyzed during this study are included in this published article.

## Abbreviations

HRQoL: Health Related Quality of Life
ICCO: Integrated Care for Childhood Overweight and obesity
CP: Coordinating Professional
PROMs: Patient Reported Outcome Measures
PedsQL4.0: Pediatric Quality of Life Inventory 4.0
IWQOL-Kids: The Impact of Weight on Quality of Life for Kids
YHC: Youth Health Care
